# Supplementary material for: Oscillating edge states in one-dimensional MoS2 nanowires
Source: Nat Commun. 2016 Oct 4;7:12904. doi: 10.1038/ncomms12904 (PMC5059444; doi:10.1038/ncomms12904)
Supplement: Supplementary Information — Supplementary Figures 1-8, Supplementary Table 1, Supplementary Discussion and Supplementary References. [file ncomms12904-s1.pdf]

SUPPLEMENTARY FIGURES

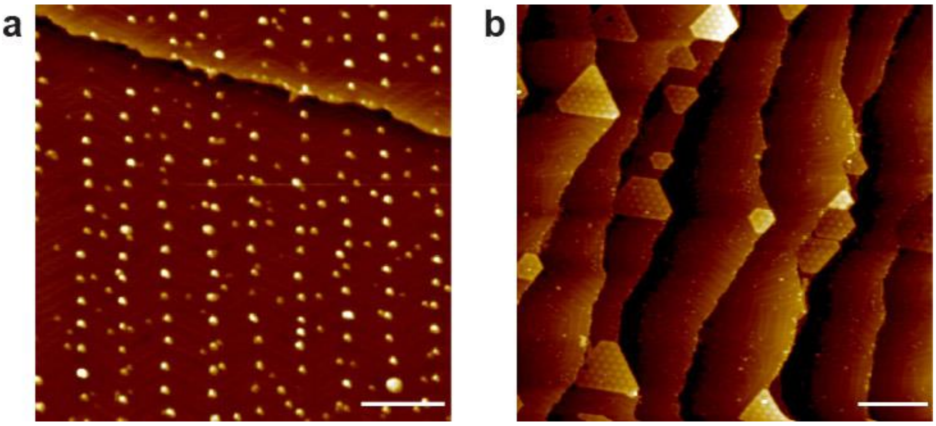

Supplementary Figure 1: **Initial stage showing monolayer MoS_2 islands formation on Au (111) surface.** **a**, Scanning tunneling microscopy (STM) image of molybdenum (Mo) clusters deposited on Au(111) surface at 100°C. The coverage of Mo clusters is about 0.06 ML (3min). **b**, STM image of the formation of monolayer MoS_2 islands after annealing (a) in H_2S at 450°C and 1×10^{-6} Torr for 40 min. Imaging parameters: ($V_b = -0.5$ V, $I_t = 800$ pA, $T = 77.8$ K). Scale bar: 20 nm.

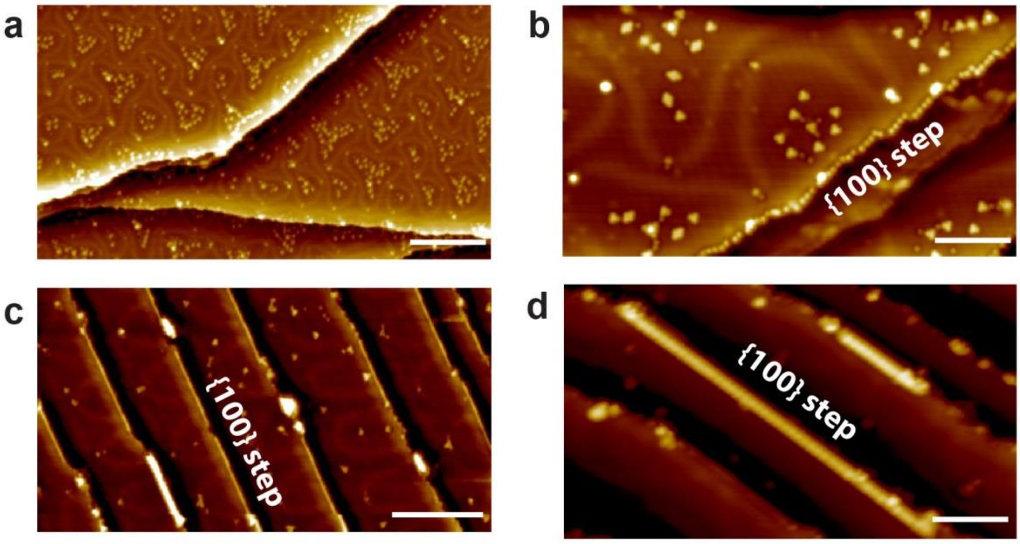

Supplementary Figure 2: **STM images of sulfur clusters and MoS_2 nanowires grown on Au (111).** **a and b**, Scanning tunneling microscopy (STM) image of sulfur deposition on clean Au (111) after exposure to H_2S at 150–180°C and 1×10^{-6} Torr for 40 min. No 1-D sulfur (S) chains can be seen at this stage, only S clusters. **c and d**, MoS_2 nanowires formed by e-beam deposition of Mo atoms for 3 min onto the Au (111) at 100°C, then annealing the Au substrate at 150–180°C in an atmosphere of

1 × 10^{-6} mbar H_2S for 40 min. It is clear that MoS_2 nanowires grow and align on the {100} step edge. Imaging Parameters: V_b = -0.5 V, I_t = 800 pA; Scale bar: **a** 20 nm, **b** 5 nm, **c** 10 nm, and **d** 5 nm, respectively.

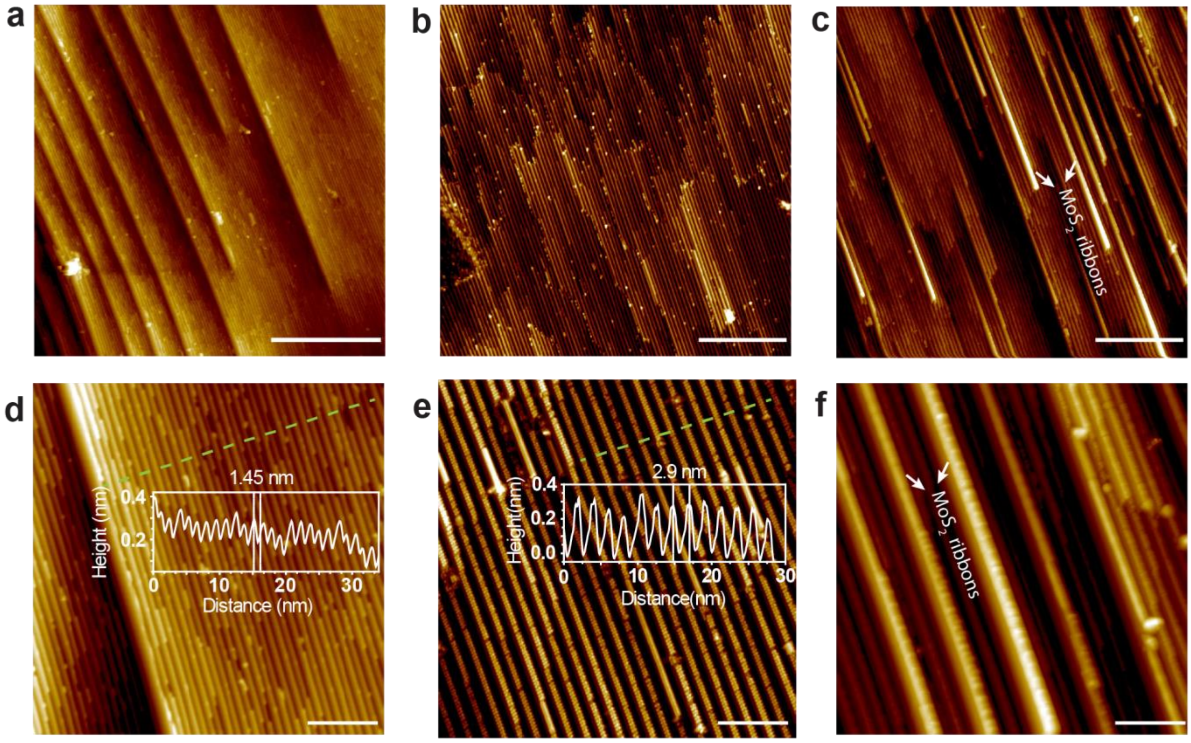

Supplementary Figure 3: **STM images of MoS_2 nanowires and nanoribbons were grown on Au (755) surface**. **a**, Bare surface cleaned by Ar^+ sputtering (V_b = −1.2 V, I_t = 400 pA). The inset shows the scanning tunneling microscopy (STM) line profile along the green line. **b**, The self-assembled MoS_2 nanowires were grown by e-beam depositing Mo for 3 min onto the Au (755) surface held at 100° C, then annealing the Au substrate at 150–180°C in an atmosphere of 1 × 10^{-6} mbar H_2S for 60 min (V_b = −1.2 V, I_t = 800 pA). The inset shows the STM line profile along the green line. **c**, STM image shows MoS_2 nanoribbon formation after sequentially annealing the MoS_2 nanowire sample (shown in **b**) at 230–250°C in an atmosphere of 1 × 10^{-6} mbar H_2S for next 40 min. The nanowires are converted to nanoribbons upon high temperature annealing. **d-f,** close-up STM images of clean Au (755) surface and MoS_2 nanostructures shown in the above panel, respectively (V_b = −1.2 V, I_t = 800 pA). Scale bar is 50 nm for **a-c**, and 10 nm for **d-e**.

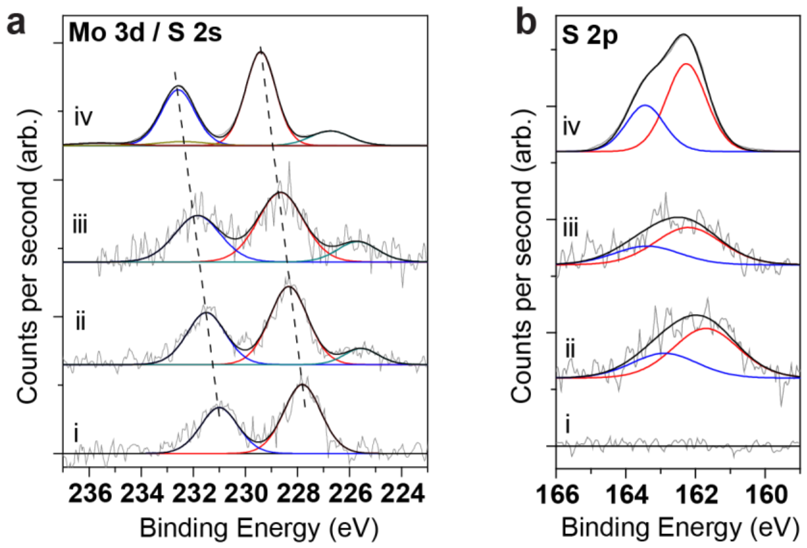

Supplementary Figure 4: **X-ray photoelectron spectra of MoS_2 nanowires grown in Au(755) surface.** Mo 3d, S 2s (**a**) and S 2p (**b**) photoelectron peaks of (**i**) Mo only, (**ii**) single MoS_2 nanowire, (**iii**) double MoS_2 nanowire on Au(755) surface. The spectra for natively cleaved MoS_2 (0001) single crystal flake (**iv**) are plotted together for comparison.

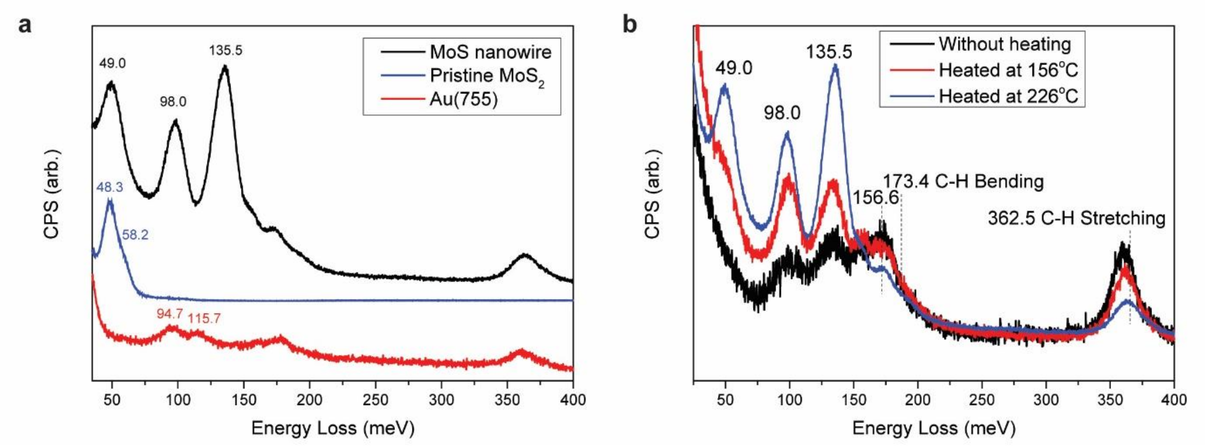

Supplementary Figure 5: **HREELS of MoS_2 nanowire.** **a**, High resolution electron energy loss spectroscopy (HREELS) spectra of MoS_2 nanowire taken in the specular direction with an incident electron energy of 4 eV. Spectrum of pristine MoS_2 single crystal is shown for comparison. **b**, HREELS spectra of MoS_2 nanowire subjected to heating at various temperatures for 1 hour as indicated, showing decrease in carbon contaminant related vibrational modes. The first three peaks increased in intensity after annealing, suggesting that they are intrinsic to the MoS_2 nanowire.

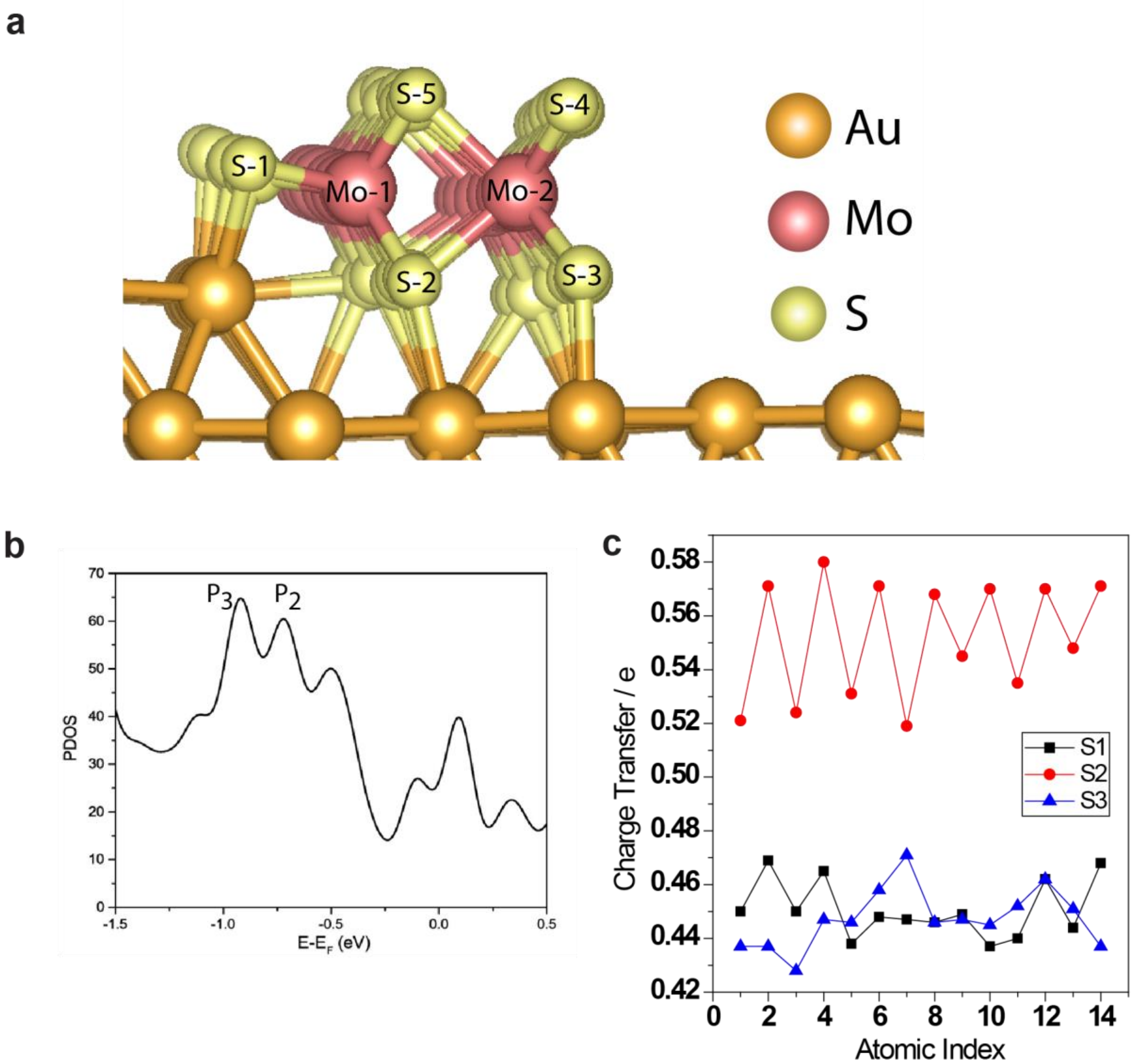

Supplementary Figure 6: **DFT calculation of the total DOS of single MoS_2 nanowire on Au(755) steps.** **a**, Schematic of the configuration of a single MoS_2 wire shown in side view (yellow atom: S, rose atom: Mo, gold atom: Au). **b**, Total density of states (DOS) and **c**, The charge transfer from the Au(755) substrate to MoS_2 nanowires. S1, S2 and S3 refer to the rows of sulfur atoms as indicated in **a.** Sulfur atoms labelled S2 gained the most charge from Au due to its position at the kink regions.

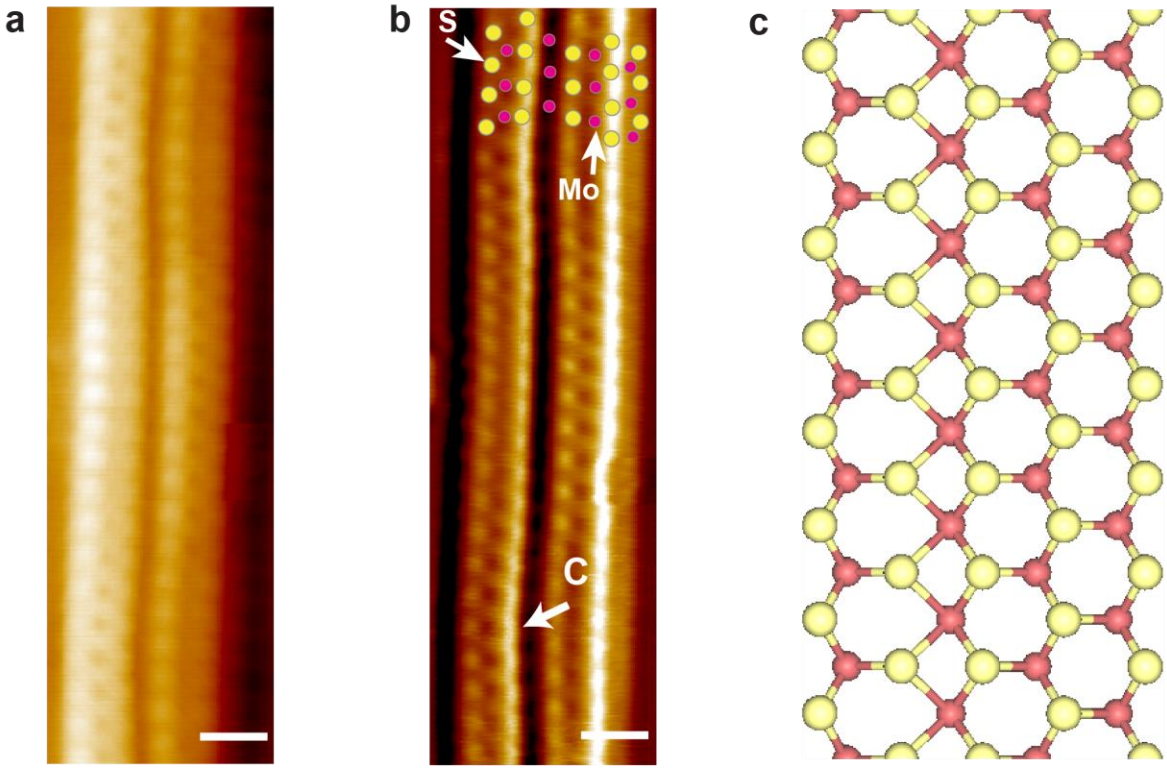

Supplementary Figure 7: **Atomic structure of twin MoS_2 nanowires grown on Au (755) surface**. **a**, Topography image of MoS_2 nanowires obtained by high resolution non-contact atomic force microscopy (NC-AFM) (f_0 = 23.1 kHz, df = −16 Hz, T = 4.5 K). **b**, The corresponding frequency image (*df* image) of MoS_2 wires, Mo(S) atom positions are marked by rose (yellow) circles at the top of the figure, and the corrugated shape is highlight by the arrow labelled as C (crest). **c**, Proposed schematic of the configuration of a twin MoS_2 wire with joined Mo boundary (yellow atom: S, rose atom: Mo). Scale bar is 0.5 nm in all NC-AFM images.

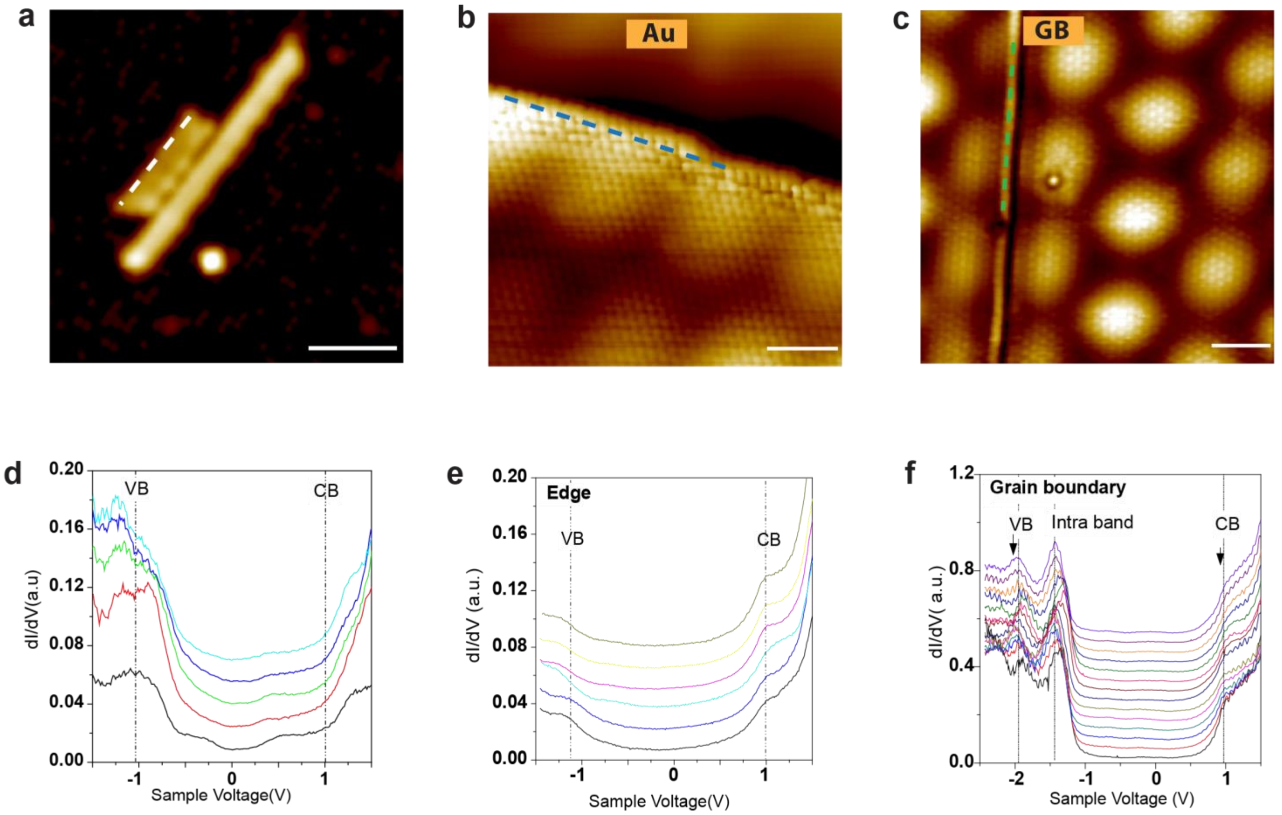

Supplementary Figure 8: **STM images and STS spectra of the edge and basal plane of MoS_2 nanoribbon and monolayer MoS_2 islands. a**, STM image of MoS_2 nanoribbon grown on Au(100) surface($V_b = -0.6$ V, $I_t = 150$ pA, $T = 77.8$ K). **b and c,** STM images of monolayer MoS_2 islands on Au(111) ($V_b = -1.0$ V, $I_t = 150$ pA, $T = 77.8$ K). **d**, STS recorded along the edge of nanoribbon (width = 2.5 nm) shown by the white dotted line in **a**. **e**, STS recorded along the edge of MoS_2 islands shown by blue dotted line in **b**. **f**, STS recorded along the grain boundary of monolayer MoS_2 islands shown by the green dotted line in **c**. It is noted that there are no periodic oscillation of the DOS or related edge states at the edge of nanoribbons, at the monolayer MoS_2 as or at the grain boundary. Scale bar is 5 nm for **a**, and 2 nm for **b** and **c**.

SUPPLEMENTARY TABLE

Sample	Mo $3d_{5/2}$	Mo $3d_{3/2}$	S 2s	S $2p_{3/2}$	S $2p_{1/2}$
Mo only	227.81	231.00			
Single wire	228.33	231.50	225.48	161.71	162.89
Twin wire	228.65	231.83	225.69	162.21	163.39
MoS_2 flake	229.41	232.58	226.74	162.27	163.45

Supplementary Table 1: **Summary of XPS results for MoS_2 nanowire grown on Au(755) surface.**

SUPPLEMENTARY DISCUSSION

1-D MoS_2 nanowires growth. Supplementary Figure 2a-b show the sulfur adsorption on Au(111) surface, in which sulfur atoms adsorbed on Au steps display sparse and discontinuous dot-like arrays. If Mo atoms were first deposited followed by sulfur atoms, aligned MoS_2 nanowires were formed along the steps under the same sulfidation condition as seen in Supplementary Fig. 2c-d. On Au (111) surface, there exists two kinds of close-packed steps with different atomic structure oriented along the <110> direction[1,2]. A descending step along the <110> presents a {111} microfacet, whereas an ascending step consists of a {100} microfacet (for simplicity, we shall refer to them as {111} and {100} steps in the text respectively). These two kinds of steps can be distinguished directly using STM[1,3]. We found that the MoS_2 nanowires prefers to grow along the {100} steps.

XPS and HREELS analysis. To shed light on the chemical bonding of the MoS_2 nanowires grown on Au surfaces, XPS was performed on samples grown with single MoS_2 nanowires, and twin MoS_2 nanowires respectively. As a comparison, the XPS spectra of pure Mo clusters prepared by e-beam evaporation on Au (755) and native cleaved MoS_2 flake were also recorded. The Mo 3d and S 2p regions of the XPS spectrum for four samples are shown in Supplementary Figure 4. All MoS_2 samples show Mo $3d_{5/2}$ binding energies (Supplementary Table 1) in the range from 228.33 to 228.65 eV. These values are chemically shifted by 0.5 eV to higher energies when compared to pure Mo, but are lower than the binding energies of stoichiometric monolayer MoS_2 and bulk MoS_2 which are in range of 229.0–229.6 eV [4,5]. The lower binding energy for Mo $3d_{5/2}$ electrons in the nanowires could be due to n-doping by the Au substrate or sulfur depletion. Upon increasing sulfur deposition, the Mo $3d_{5/2}$ peak shifts to a higher binding energy which is closer to the values of bulk MoS_2. For the twin MoS_2 nanowire samples, the Mo $3d_{5/2}$ peak shifts to higher binding energy closer to native MoS_2 position. The S $2p_{3/2}$ peaks are observed at 161.71–162.21 eV, which is consistent with the value of 162.1 eV for monolayer MoS_2 grown on Au surface[4,6].

In Supplementary Figure 5, signature MoS_2 A_1 phonons were observed in pristine 2D MoS_2 monolayer at 47.7 and 58.2 meV, which is consistent with literature values[7]. In comparison, MoS_2 nanowire exhibits three modes at 49.0, 98.0 and 135.5 meV. These peaks do not originate from the Au (755) substrate as shown in Figure S5a. The additional modes at 94.7 and 115.7 meV belong to the C-H rocking mode. We have heated the sample to various temperatures as shown in Figure S5b to remove contaminations. The peak at 49.0 meV can be assigned to the Mo-S stretch[8,9]. The 49.0 meV peak agrees with the surface phonon of MoS_2, which is the basic repeating unit of our MoS_2 nanowire backbone. We assign the 98.0 and 135.5 meV peaks to the edge atoms on both asymmetric

ends of the MoS_2 nanowires. The decreased coordination and reduced symmetry of atoms on stepped surfaces, in contrast to those in the bulk material and on Au terraces, lead to bond stiffening. The shrinkage in bond length between the edge atoms and bulk atoms is accompanied by a stiffening of the force constant between these two atoms in the direction perpendicular to the macroscopic surface.

***Ab initio* calculations.** Our first-principles electronic structure calculations are based on density functional theory (DFT) using Vienna *ab initio* Simulation Package(VASP)[10]. DFT calculations were performed with a plane wave basis (350 eV for the kinetic energy cutoff) and a 1×1×1 k-point sampling of Brillouin zone. In all calculations, the local spin density approximation (LSDA) and generalized gradient approximation (GGA) in Perdew-Burke-Ernzerhof (PBE) format[11] together with the projector-augmented-wave (PAW) method[12] was included. In the supercell, a vacuum region of 11.6 Å in the direction normal to the Au surface (z direction) was used to avoid interactions between two neighboring cells. In optimizing atomic structure, the force convergence criterion was set to 0.01 eV $Å^{-1}$. As we known, DFT significantly underestimates bandgaps of semiconducting materials by 50% due to the neglect of strong correlations in XC functionals. However, MoS_2 is an exceptional case that DFT is able to generate reasonably good bandgap. For a monolayer MoS_2, a previous DFT study gave a bandgap around 1.7 eV, agreeing very well with the experimental value of 1.8[13]. Our DFT calculations yielded 1.68 eV of the bandgap of a monolayer MoS_2 (see figure below for the DOS), perfectly matching previous results.

STS measurement. To investigate the effect of system size on the oscillation of the edge states, STS was carried out on the edges of the wider MoS_2 nanoribbons and large monolayer MoS_2 islands grown on Au(100) and Au (111) surface respectively, as shown in Supplementary Figure 9a-f. STS spectra recorded on the edge of MoS_2 nanoribbons, the valence band (VB) is located at around –1.0 eV, close to the edge state observed in MoS_2 nanowires and narrow nanoribbons (width < 2nm). No obvious oscillation of the edge state is observed, unlike that of the nanowire discussed in the manuscript.

SUPPLEMENTARY REFERENCES

1 Rousset, S., Repain, V., Baudot, G., Garreau, Y. & Lecoeur, J. Self-ordering of Au(111) vicinal surfaces and application to nanostructure organized growth. *Journal of Physics: Condensed Matter* **15,** S3363 (2003).

2 Biener, M. M., Biener, J. & Friend, C. M. Revisiting the S-Au(111) interaction: static or dynamic? *Langmuir* **21,** 1668-1671 (2005).

3 V.Repain, J. M. B., S.Rousset and J. Lecoeur. Interaction between steps and reconstrcution on Au(111). *Europhys. Lett.* **47,** 435-441 (1999).

4 Sorensen, S. G., Fuchtbauer, H. G., Tuxen, A. K., Walton, A. S. & Lauritsen, J. V. Structure and electronic properties of in situ synthesized single-layer MoS2 on a gold surface. *ACS Nano* **8,** 6788-6796 (2014).

5 Mak, K. F., Lee, C., Hone, J., Shan, J. & Heinz, T. F. Atomically Thin MoS_2: A New Direct-Gap Semiconductor. *Physical Review Letters* **105,** 136805 (2010).

6 Zeng, C., Kent, P. R. C., Kim, T.-H., Li, A.-P. & Weitering, H. H. Charge-order fluctuations in one-dimensional silicides. *Nat Mater* **7,** 539-542 (2008).

7 Peierls, R. E. *Quantum Theory of Solids*. (Oxford Univ. Press, Oxford, 1955).

8 Weber, T., Muijsers, J. C., van Wolput, J. H. M. C., Verhagen, C. P. J. & Niemantsverdriet, J. W. Basic Reaction Steps in the Sulfidation of Crystalline MoO_3 to MoS_2, As Studied by X-ray Photoelectron and Infrared Emission Spectroscopy. *The Journal of Physical Chemistry* **100,** 14144-14150 (1996).

9 Chang, C. H. & Chan, S. S. Infrared and Raman studies of amorphous MoS_3 and poorly crystalline MoS_2. *J. Catal.* **72,** 139-148 (1981).

10 Kresse, G. & Furthmüller, J. Efficient iterative schemes for *ab initio* total-energy calculations using a plane-wave basis set. *Physical Review B* **54,** 11169-11186 (1996).

11 Perdew, J. P., Burke, K. & Ernzerhof, M. Generalized Gradient Approximation Made Simple. *Physical Review Letters* **77,** 3865-3868 (1996).

12 Blöchl, P. E. Projector augmented-wave method. *Physical Review B* **50,** 17953-17979 (1994).

13 Feng, N. *et al.* First Principles Prediction of the Magnetic Properties of Fe-X6 (X = S, C, N, O, F) Doped Monolayer MoS2. *Scientific Reports* **4,** 3987 (2014).
